# Does integrated health management within a county medical consortium improve rural type 2 diabetic patients’ self-management behavior and quality of life? An empirical analysis from Eastern China

**DOI:** 10.1186/s12889-024-18885-0

**Published:** 2024-05-29

**Authors:** Mingyao Peng, Li Li, Xinyi Shi, Zhonghua Wang

**Affiliations:** 1 School of Health Policy & Management, Nanjing Medical University, Nanjing, 211166 China; 2 Binhai County People’s Hospital, Yancheng, 224500 Jiangsu China; 3 The Public Health Policy and Management Innovation Research Team, Nanjing Medical University, Nanjing, 211166 China; 4 School of Public Health, Nanjing Medical University, Nanjing, 211166 China

**Keywords:** Self-management behavior, Quality of life, Type 2 Diabetes, Medical consortium, Integrated health management

## Abstract

**Background:**

Levels of self-management behaviors (SMB) and quality of life (QoL) are suboptimal in Chinese adults with type 2 diabetes (T2D), especially in rural China. Integrated health management within a county medical consortium, featuring multi-level teams of doctors, nurses, and other professionals offering follow-up services such as check-ups, assessments, treatment, and health education, is promising in improving this. This study aimed to assess the effect of integrated health management within a county medical consortium on the SMB and QoL of rural T2D patients in China.

**Methods:**

Based on a survey conducted on the county medical consortium in Eastern China, this study initially employed propensity score matching (PSM), a nonparametric technique, to precisely estimate the average treatment effect on the treated (ATT) of integrated health management on SMB and QoL in rural T2D patients. Subsequently, quantile regression was also performed to estimate the relationship between the implementation of integrated health management, sociodemographic factors, follow-up services (offered during integrated health management) and both SMB and QoL.

**Results:**

The ATT values for SMB and QoL, representing the net effect of integrated health management within a county medical consortium on SMB and QoL, were significantly positive. They ranged from 4.34 to 4.67 for SMB and from 0.89 to 1.06 for QoL, respectively, based on the four different PSM modalities. The results of quantile regression also revealed a statistically significant positive association between the implementation of integrated health management and both SMB (coef. = 4.15) and QoL (coef. = 1.54). These findings suggest that integrated health management within a county medical consortium can effectively improve SMB and QoL in rural T2D patients. Furthermore, frequency of follow-up service and health behavior guidance were positively associated with SMB and QoL. Conversely, on-call follow-up services, medication guidance and follow-up services at medical institutions were negatively correlated with SMB or QoL.

**Conclusions:**

The study highlights the effectiveness of integrated health management within a county medical consortium in improving SMB and QoL among individuals with T2D in rural China. The findings offer invaluable insights for the advancement of chronic disease management in rural areas of developing countries.

**Supplementary Information:**

The online version contains supplementary material available at 10.1186/s12889-024-18885-0.

## Introduction

Diabetes is currently one of the most widespread and life-threatening chronic illnesses worldwide. The global prevalence of diabetes among adults aged 20—79 years was estimated at 9.8% (536.6 million) in 2021; this is predicted to reach 12.2% (783.2 million) by 2045 [1]. Notably, China had the highest number of diabetic individuals in 2021, with a prevalence of 10.6% (total of 141 million individuals, of which more than 90% have type 2 diabetes (T2D)) [1]. Moreover, 78% of Chinese adults with T2D develop several complications [2]. Patients’ physical and psychological health is significantly impacted by diabetes and its complications, which also poses a significant challenge for healthcare and society [3]. Self-management behavior (SMB) refers to the daily behaviors that diabetic individuals adopt to manage their condition. These behaviors include self-monitoring of blood glucose, medication adherence and compliance, dietary, exercise activities, and foot care, among others; the aim is to monitor symptoms and maintain and improve health outcomes. The World Health Organization (WHO) has emphasized that improving diabetes patients’ self-management ability is more effective than any other intervention measure in disease control [4]. National and international studies have consistently demonstrated suboptimal levels of SMB among individuals with T2D. In the United States, only 42% meet dietary guidelines while 70% fall short in physical activity [5]. Similarly, Australian T2D patients attained an average 50% self-management score, with low performance in exercise and foot care [6]. Several studies from developing countries have also revealed similar results [7, 8]. In China, T2D patients have demonstrated poor adherence to self-monitoring of blood-glucose and foot care [3]. Notably, owing to the considerable urban-rural gap in terms of economic and social development, T2D patients in rural China tend to have lower health literacy, poorer living environments, and lower quality of healthcare, resulting in poorer health behaviors and quality of life (QoL) [9].

Interventions for management of diabetes may improve patient SMB and QoL. Studies have demonstrated that support from peers, medical professionals, or health education interventions may enhance T2D patients’ SMB and improve their QoL [10, 11]. However, the impact of these interventions is limited by a fragmented and intermittent nature of disease management and treatment, addressing the patients’ wide-ranging and comprehensive needs inadequately, and ultimately resulting in a limited impact in improving SMB and health outcomes [12]. The WHO first proposed integrated health care in 1996 [13], marking the advent of a more systematic approach to managing chronic conditions such as T2D. This approach was further developed through models like the Chronic Care Model by Ed Wagner and the Innovative Care for Chronic Conditions (ICCC) [14, 15]. By connecting services from multiple healthcare providers, integrated health management helps boost service continuity while improving health behaviors and outcomes in patients with chronic conditions. These models have been successfully implemented in Australia, Russia, and other countries [16–19]. Studies show that patients with chronic conditions can benefit from it in terms of both health behaviors and outcomes [20–22]. In 2016, the WHO formally defined integrated health management as “a continuum of health promotion, disease prevention, diagnosis, treatment, disease management, rehabilitation, and palliative care services for people, coordinated at different levels and locations within and outside the health sector”, and emphasizes the importance of integrated health management in guiding people with chronic diseases to participate in self-management [23].

Promoting integrated health management within a medical consortium has emerged as a crucial focus of the ongoing healthcare system reform in China. In 2017, the General Office of the State Council issued “Guidance on Promoting the Construction and Development of Medical Consortium”, emphasizing the pivotal role of medical consortium for advancing healthcare reform. In 2020, the National Health Commission issued the “Management Measures for Medical Consortium” with the aim of expediting the establishment of integrated health management within the medical consortiums. The medical consortium mainly comprises urban and county-level consortiums. Urban medical consortiums are in cities and are led by tertiary public hospitals; they are formed through collaboration with community health service institutions, nursing homes, and professional rehabilitation centers. County consortiums are composed of three levels of medical care institutions in rural areas, led by a county-level hospital; township health centers act as hubs and village health offices act as bases. Integrated health management within a county medical consortium is provided by teams that combine doctors, nurses, and other healthcare professionals from various levels including county, township, and village. The teams provide follow-up services that include routine check-ups, health assessments, disease diagnosis and treatment, and health education, among others. One of the key goals of integrated health management within a county medical consortium is to help patients with chronic disease achieve better health behaviors and outcomes; it therefore offers a promising model for improving SMB and QoL in T2D patients. Over the past few years, several studies have assessed the effect of integrated health management within urban medical consortiums in China. These studies found these consortiums were effective in improving health behaviors and outcomes in individuals with chronic diseases [24, 25]. However, these studies only reported a correlation between integrated health management and health behaviors and outcomes using regression analysis; they have not provided a precise estimate of the policy effects of such management approaches on health behaviors and outcomes. In addition, prior research has not evaluated the impact of integrated health management within a county medical consortium on health behaviors and outcomes among rural patients with chronic disease.

Due to significant urban-rural disparity in China, T2D patients from rural areas tend to have lower health literacy, poorer living environments, and lower quality of healthcare, resulting in poorer SMB and QoL. Integrated health management within a county medical consortium provides a viable model for improving SMB and QoL among rural T2D patients. However, it remains unclear whether integrated health management affects SMB and QoL in rural patients with T2D. This study initially utilized propensity score matching (PSM), a nonparametric technique, to estimate the average treatment effect on the treated (ATT) of integrated health management within a county medical consortium on the SMB and QoL of rural T2D patients. PSM was employed to control for confounding bias originating from observable variables in the treatment and control groups, ensuring accurate assessment of the net impact of policy implementation. Subsequently, quantile regression was additionally performed to estimate the relationship between the implementation of integrated health management, sociodemographic factors, follow-up services (offered during integrated health management) and both SMB and QoL in rural T2D patients.

## Methods

### Data source

The data used in this study were obtained from a survey on integrated health management within the county medical consortium of Binhai County, Jiangsu Province, eastern China. In 2019, 24 counties in Jiangsu Province were selected as pilot areas for closely integrated county medical communities. As one of the pilot areas, Binhai county developed a typical county medical consortium, which is led by Binhai County People’s Hospital; the Caiqiao Township Health Center serves as the hub and village health offices serve as the bases. Residents are provided integrated health management by health management teams, which comprise doctors and nurses from medical institutions of different levels within the county medical consortium. Health management teams regularly provide follow-up services for T2D patients, monitoring their health status and providing timely interventions. The diabetes follow-up service encompasses blood-glucose monitoring, inquiry about diabetes symptoms, medication guidance, health behavior guidance, and treatment for a condition other than diabetes. The teams deliver services in households or on-call, and patients can also visit medical institutions for follow-up services.

We included T2D patients from Caiqiao Town (which implemented integrated health management within the county medical consortium) in the treatment group; patients from Zhenghong Town were included in the control group (a town is geographically adjacent, but outside the county medical consortium and does not implement integrated health management). The survey was conducted in May 2022. All patients were enrolled by whole-group sampling using the township health center database, and the survey was conducted by surveyors in collaboration with family physicians. Inclusion criteria for participants encompassed the following demographic characteristics: rural residential registration; the age of 18 years or older; and confirmed T2D diagnosis as per medical records. Exclusion criteria comprised adults with cognitive impairments and mental disorders affecting communication (e.g., aphasia or deafness), which could potentially hinder survey completion. Questionnaire content included patients’ basic information, socioeconomic characteristics, follow-up services for treatment group (frequency, mode, and content), self-efficacy, social support, diabetes SMB, and QoL. A total of 2,193 questionnaires were completed, of which 1,792 were deemed valid (890 in the treatment group and 902 in the control group), with an effective rate of 81.71%.

### Variable selection

The dependent variables were QoL and SMB, and the independent variables included the implementation of integrated health management, the follow-up service (frequency, mode, and content), self-efficacy, social support, diabetes-related knowledge, diabetes-related distress, and demographic characteristics (age, gender, personal income, education, marriage, employment, co-morbidity, incapacity, and self-reported health). Table 1 provides a detailed description of the variables.

**Table 1:** Definition of variables

Variable	Description	Indicators/survey questions
**Dependent variables**		
Quality of life (QoL)	162 – (Sum of D-QoL)	Twenty-seven questions of the D-QoL
Physiology	6 – (Mean of physiology dimension)	Physiology dimension of D-QoL (items 1-12)
Psychology	6 – (Mean of psychology dimension)	Psychology dimension of D-QoL (items 13-20)
Social	6 – (Mean of social dimension)	Social dimension of D-QoL (items 21-24)
Therapy	6 – (Mean of therapy dimension)	Therapy dimension of D-QoL (items 25-27)
Self-management (SM)	Sum of SDSCA	Eleven questions of the SDSCA
General diet	Mean of general diet dimension	General diet dimension of SDSCA (items 1-2)
Specific diet	Mean of specific diet dimension	Specific diet dimension of SDSCA (items 3-4)
Exercise	Mean of exercise dimension	Exercise dimension of SDSCA (items 5-6)
Blood-glucose testing	Mean of blood-glucose testing dimension	Blood-glucose testing dimension of SDSCA (items 7-8)
Foot care	Mean of medications dimension	Medications dimension of SDSCA (items 9-10)
Medications	Mean of foot care dimension	Foot care dimension of SDSCA (item 11)
**Independent variables**		
Implement of integrated health management	=1, if yes; =0, if not no	
Follow-up service frequency	=1, if >2; =0, if ≤2	How many times did you receive follow-up service from the integrated health management team in the last three months?
Follow-up service content		
Measuring blood glucose	=1, if acquired; =0, if not acquired	Question: What are the components of the follow-up service you received from the integrated health management team?
Inquiring about diabetes symptoms	=1, if acquired; =0, if not acquired	
Medication guidance	=1, if acquired; =0, if not acquired	
Treatment for a condition other than diabetes	=1, if acquired; =0, if not acquired	
Health behavior guidance	=1, if acquired; =0, if not acquired	
Follow-up service mode		
Households and home visiting	=1, if acquired; =0, if not acquired	Question: What are the modes of follow-up services you received from the integrated health management team? (Multiple choice)
Going to a medical institution	=1, if acquired; =0, if not acquired	
On call	=1, if acquired; =0, if not acquired	
Self-efficacy	Mean of SECD6	Six questions of the SECD6
Support from family and friends	Mean of the family/friend subscales	The family/friend subscales of the CIRS
Support from physician/health care team	Mean of the doctor/health care team subscales	The doctor/health care team subscales of the CIRS
Support from neighborhood/community	Mean of the neighborhood/community subscales	The neighborhood/community subscales of the CIRS
Diabetes-related knowledge	Number of questions properly answered / Number of questions answered	Questions of the AD-knowl
Diabetes-related distress	Sum of PAID questions	Twenty questions of the PAID
Age (year)	=1, if ≥65; =0, if <65	Question: Year of birth
Gender	=1, if female; =0, if male	Question: What is your sex?
Personal income ($)	=1, if ≤149; =2, if >149 and ≤297; =3, if >297 and ≤743;=4, if >743	Question: What was your total income in the previous year?
Education	=1, if elementary school and below; =2, junior high school; =3, if high school and above	Question: What is your education level?
Marital status	=1, if married; =0, if single, widowed, divorced, and other	Question: What is your marital status?
Employment status	=1, if unemployed; =2, if employed; =3, if retire	Question: What is your employment status?
Co-morbidity	=1, if having chronic diseases other than diabetes; =0, if diabetes only	Question: Have you been diagnosed with any chronic diseases?
Incapacity	=1, if without incapacity; =2, if with incapacity	Question: Do you have any incapacity (such as dressing/undressing, dining, and bathing, among others)
Self-reported health	Continuous variables	Question: How would you rate your health today on a scale of 0 (worst possible health) to 100 (best possible health)? (EQ-VAS)

*SECD6* Self-Efficacy for Managing Chronic Disease 6-Item Scale, *D-QoL* Diabetes-specific Quality-of-Life, *SDSCA* Summary of Diabetes Self-Care Activities, *CIRS* Chronic Illness Resources Survey, *AD-knowl* Audit of Diabetes Knowledge, *PAID* Problem Areas in Diabetes Scale, *EQ-VAS* EuroQol Visual Analogue Scale

The scales of the relevant variables used in this study were as follows:

The Diabetes-specific Quality-of-Life (D-QoL)

The D-QoL was widely used for assessing the QoL of T2D patients [26]. The scale has four dimensions (physiology, psychology, social, and therapy) and 27 components in the Chinese version and has been tested for reliability and validity [26]. Scores for the responses to each item range from 1 (very satisfied) to 5 (very dissatisfied); the items are scored in reverse order. We converted the scores for each item to a positive score to facilitate the interpretation of the results; this score was obtained by adding 1 to the difference between the maximum and reverse score (Table 1).

The Summary of Diabetes Self-Care Activities (SDSCA)

Toobert et al. created the SDSCA tool to assess SMB in T2D patients. This study used the SDSCA with 6 dimensions (11 items): food management, foot care, glucose monitoring, exercise management, and medication management [27]. The response to each item indicates the number of days the patient performed the behavior during the previous week. The SDSCA is currently the most widely used and authoritative scale to measure the SMB of diabetic patients. The Chinese version of the SDSCA scale was utilized in this study. Qiao et al. localized and tested the original SDSCA scale, and the internal consistency reliability of the Chinese version of the SDSCA scale was 0.918 [28].

Additionally, self-efficacy was evaluated using the Self-Efficacy for Managing Chronic Disease 6-Item Scale (SECD6). Multi-social support measures were assessed using the Chronic Illness Resources Survey (CIRS), while diabetes knowledge was gauged with the Audit of Diabetes Knowledge (AD-knowl). Diabetes-related distress was quantified using the Problem Areas in Diabetes Scale (PAID), and self-reported health was measured via the EuroQol Visual Analogue Scale (EQ-VAS). All scales were utilized in their Chinese versions and were validated in Mandarin. The detailed descriptions of these scales are provided in the supplementary materials.

### Statistical analysis

#### Descriptive analysis

Data were analyzed using STATA 16.0 (College Station, TX, USA). To obtain a preliminary overview of the characteristics, SMB, and QoL among T2D patients, we calculated the median and interquartile range (IQR) for continuous variables and the prevalence and 95% credibility intervals (CI) for categorical variables.

#### Propensity score matching

Propensity score matching (PSM) is a suitable nonparametric method for estimating the net effect of policy implementation, as it allows for control of confounding bias arising from observable variables between treatment and control groups; it also enables accurate estimation of the ATT of policy implementation [29]. Therefore, we conducted PSM to precisely estimate the ATT of integrated health management within the county medical consortium on SMB and QoL of rural patients with T2D.

In the matching process, a binary dummy variable T was used, where T=1 represented the treatment group and T=0 represented the control group. To avoid confounding bias, the matching approach identified patients in the control group who had very similar likelihood of obtaining integrated health management as patients in the treatment group. The following formula reflected their likelihood of obtaining integrated health management:

$${P}_{i}(X)=\text{Pr}\left\{A=T\right\}=F\{h({X}_{i})\}$$

${X}_{i}$ was the matching variable that signified the characteristic variable for the *i*th T2D patient. We selected self-efficacy, social support, diabetes-related knowledge, diabetes-related distress, age, gender, personal income, education, marriage, employment, co-morbidity, incapacity, and self-reported health as matching variables based on previous studies and the law of maximizing R^2^; $h(. )$ indicated a linear function, $F\left(.\right)$ a logit function, and ${P}_{i}(X)$ the predicted probability value. We used K-nearest neighbor matching, caliper specification, and kernel matching to address the constraints of continuous variable propensity score estimation [30]. K values of 1 and 4 for the K-nearest neighbor matching and a 0.02 caliper tolerance were used to restrict the absolute variance of PSM for an observation pair.

Based on the above model, we initially performed the test of the common support assumptions to ensure that the treatment and control groups have a common support across all covariates. Then we performed the covariate imbalance test for the treatment and control groups to guarantee the control for potential confounding bias originating from observable variables. Subsequently, the ATT for integrated health management was calculated. In order to test the robustness of the PSM results, we estimated the ATT using four different PSM modalities (1:4 nearest neighbor matching, 1:4 intra-caliper nearest neighbor matching, 1:1 nearest neighbor matching, and kernel matching).

#### Quantile regression

The quantile regression, akin to the classical least squares approach in linear regression, involves minimizing the absolute residuals asymmetrically. This method becomes especially relevant when the distribution of explanatory variables deviates from normality, rendering traditional least squares estimation ineffective. In such cases, quantile regression provides a more robust alternative for statistical analysis. In this study, the choice to utilize quantile regression was driven by the non-normal distributions of QoL and SMB, necessitating a more flexible estimation method.

After matching the samples in the treatment and control groups, the sample frequencies derived from PSM can be utilized as weighting factors to perform a weighted regression analysis on the relevant factors. This approach facilitates the adjustment for potential confounders, thereby strengthening the statistical validity and precision of the regression analysis. We utilized frequency-weighted quantile regression to explore the association of the implementation of integrated health management, sociodemographic factors with QoL and SMB. In addition, general quantile regression was also employed to investigate the relationship between follow-up services (offered during integrated health management) and both QoL and SMB in the treatment group.

## Results

### Characteristics of the sampled diabetic patients

Table 2 provides a detailed description of the participant characteristics in the treatment (890 individuals) and control (902 individuals) groups. In the treatment group, 65.3% of the respondents were aged 65 years old and above, 37.2% were male, 83.3% were married, and 48.5% had an income of > $297 per year. Most respondents had an education level of elementary school and below (76.1%) and were unemployed (66.3%); 67.2% of respondents had a chronic disease other than diabetes. The median QoL value was 116.00; the physiology dimension had the lowest median value (4.17), and the social dimension had the highest median value (4.75). The median value of SMB was 40.00; medication had highest median value at 7.00. Blood-glucose measurement and foot care had lower median values of only 2.00 and 1.50, respectively. Approximately 80.2% of respondents received follow-up services for more than twice in the past 3 months; most received blood glucose measurement (99.3%), inquiry about diabetic symptoms (87.8%), treatment for a condition other than diabetes (63.6%), medications guidance (83.1%), and health behavior guidance (64.6%). Follow-up services were mainly implemented in the form of household and home visits (99.2%) and visits to a medical institution (81.3%).

**Table 2:** Descriptive statistics of the sampled rural T2D patients

Variable	Total (*n*=1,792)		Treatment group (*n*=890)		Control group (*n*=902)	
	Median / n	IQR / Proportion	Median / n	IQR / Proportion	Median / n	IQR / Proportion
Quality of life (QoL)	114.00	11.00	116.00	12.00	112.00	10.00
Physiology	4.08	0.67	4.17	0.58	3.92	0.58
Psychology	4.25	0.50	4.38	0.63	4.13	0.50
Social	4.75	0.50	4.75	0.50	4.75	0.25
Therapy	4.33	0.67	4.50	0.33	4.33	0.67
Self-management (SM)	38.00	10.00	40.00	10.00	36.00	8.00
General diet	5.50	1.50	6.00	1.50	5.50	1.50
Specific diet	5.50	1.00	5.50	1.50	5.00	1.00
Exercise	2.50	2.50	3.00	3.00	2.00	2.50
Blood-glucose testing	1.00	2.00	2.00	1.00	1.00	1.13
Medications	7.00	1.00	7.00	0.00	7.00	1.00
Foot care	0.00	2.50	1.50	3.00	0.00	1.50
Follow-up management frequency						
≤2	176	9.8%	176	19.8%	—	—
>2	713	39. 8%	713	80.2%	—	—
Follow-up management content						
Measuring blood glucose	884	49.3%	884	99.3%	—	—
Inquiring about diabetes symptoms	775	43.2%	775	87.1%	—	—
Medication guidance	740	41.3%	740	83.1%	—	—
Treatment for a condition other than diabetes	566	31.6%	566	63. 6%	—	—
Health behavior guidance	575	32.1%	575	64.6%	—	—
Follow-up management mode						
Households and home visiting	883	49.3%	883	99.2%	—	—
Going to a medical institution	724	40.4%	724	81.3%	—	—
On call	340	19.0%	340	38.2%	—	—
Self-efficacy	6.33	1.67	6.67	1.67	6.17	1.50
Support from physician/health care team	4.25	0.75	4.25	0.50	4.25	0.75
Support from family and friends	3.80	0.80	3.80	0.80	3.80	0.80
Support from neighborhood/community	3.00	1.33	3.00	1.33	3.00	1.00
Diabetes-related knowledge (%)	52.08	18.75	51.04	16.67	53.47	18.07
Diabetes-related distress	11.00	9.00	9.00	12.00	12.00	9.00
Age (year)						
<65	584	32.6%	309	34.7%	275	30.5%
≥65	1208	67.4%	581	65.3%	627	69.5%
Gender						
Male	665	37.1%	331	37.2%	334	37.0%
Female	1127	62. 9%	559	62.8%	568	63.0%
Personal income ($)						
≤149	199	11.1%	126	14.2%	73	8.1%
>149 and ≤297	766	42.7%	332	37.3%	434	48.1%
>297 and ≤743	455	25.4%	232	26.1%	223	24.7%
>743	372	20.8%	200	22.4%	172	19.1%
Education						
Elementary school and below	1337	74.6%	677	76.1%	660	73.2%
Junior high school	319	17.8%	146	16.4%	173	19.2%
High school and above	136	7.6%	67	7.5%	69	7.6%
Marital status						
Single, widowed, divorced, or other	295	16.5%	149	16.7%	146	16.2%
Married	1202	83.6%	741	83.3%	756	83.8%
Employment status						
Unemployed	1121	62. 6%	590	66.3%	531	58. 9%
Employed	623	34.8%	271	30.4%	352	39.0%
Retired	48	2.7%	29	3.3%	19	2.1%
Co-morbidity						
Diabetes only	612	34. 2%	292	32.8%	320	35.5%
Having chronic diseases other than diabetes	1180	65.8%	598	67.2%	582	64.5%
Incapacity						
Without incapacity	1636	91.3%	821	92.2%	815	90.4%
Incapacity	156	8.7%	69	7.8%	87	9.6%
Self-reported health	70.00	11.00	70.00	15.00	70.00	5.00

*IQR* Interquartile range

In the control group, 69.5% of respondents were aged at least 65 years, 37.0% were male, 83.8% were married, and 48.1% had an income level of $149-297. Most respondents had an education level of elementary school and below (73.2%) and were unemployed (58.9%); 64.5% had a chronic disease other than diabetes. The median QoL score was 112.00; the physiology dimension had the lowest median value (3.92) and the social dimension had the highest (4.75). The median value of SMB was 36; the median values for blood-glucose measurement and foot care were lower at 1.00 and 0.00, respectively, and medication had the highest median value at 7.00.

### Propensity score matching results

**Fig. 1:**
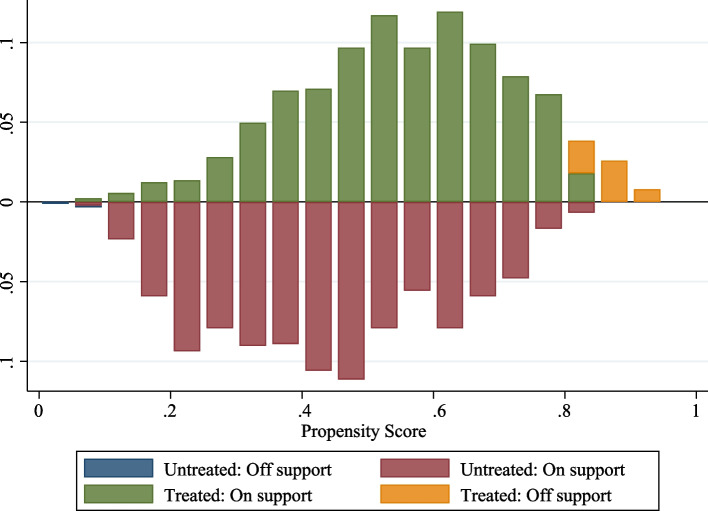
Propensity score histogram

Figure 1 displays the histogram of propensity scores, which exhibits a similar distribution for both groups. Specifically, the propensity score of T2D patients in the treatment group were primarily concentrated within the 0.30-0.80 range; for the control group, it was mainly concentrated in the range of 0.20-0.70. Only a small number of patients were off support. The significant overlap between the propensity scores of the treated and control groups provides evidence supporting the common support assumption.

**Fig. 2:**
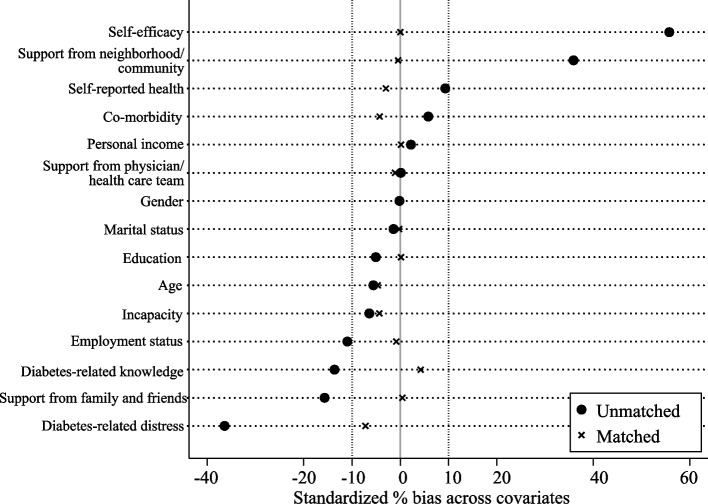
Standardized percentage bias across covariates (1:4 nearest neighbor matching)

Table 3 and Fig. 2 present the results of covariate imbalance testing for1:4 nearest neighbor matching, both statistically and graphically. Before matching, the covariates of self-efficacy, diabetes-related knowledge and support from neighborhood/community had absolute values of standardized percentage biases greater than 20.0%. Furthermore, variables such as self-efficacy, support from physician/health care team, support from neighborhood/community, diabetes-related distress, diabetes-related knowledge, employment status, and self-reported health were found to differ significantly (*p*-values < 10%) between the groups. After matching, the absolute values of the standardized percentage biases declined for all covariates, and all *p*-values were greater than 10%, indicating no significant difference between the treatment and control groups and confounding bias arising from observable variables has been controlled. The results of covariate imbalance testing for other three PSM modalities were similar (Table S1-S3 and Figure S1-S3 see Additional file 1).

**Table 3:** Covariate imbalance testing (1:4 nearest neighbor matching)

Variable	Unmatched				Matched			
	Treated	Untreated	%Bias	*P* value	Treated	Untreated	%Bias	*P* value
Self-efficacy	6.71	6.00	55.7	<0.001***	6.59	6.59	0	0.997
Support from family and friends	4.23	4.23	0.1	0.981	4.22	4.23	-1.1	0.816
Support from physician/health care team	3.72	3.82	-15.7	0.001***	3.72	3.72	0.4	0.928
Support from neighborhood/community	3.08	2.75	35.9	<0.001***	3.03	3.04	-0.4	0.925
Diabetes-related distress	10.36	13.11	-36.4	<0.001***	10.75	11.30	-7.2	0.128
Diabetes-related knowledge	50.55	52.78	-13.6	0.004***	51.02	50.32	4.2	0.326
Age	1.63	1.63	-0.2	0.972	1.63	1.63	-0.4	0.94
Gender	67.40	67.87	-5.6	0.239	67.47	67.87	-4.7	0.33
Personal income	2.57	2.55	2.2	0.641	2.57	2.57	0.2	0.974
Education	1.32	1.35	-5.1	0.284	1.32	1.32	0.1	0.976
Marital status	0.83	0.84	-1.4	0.767	0.83	0.83	-0.2	0.961
Employment status	1.37	1.43	-11	0.021**	1.38	1.38	-0.8	0.863
Co-morbidity	0.67	0.65	5.8	0.22	0.67	0.69	-4.3	0.373
Incapacity	0.08	0.10	-6.4	0.175	0.08	0.09	-4.3	0.363
Self-reported health	73.31	72.23	9.3	0.049**	72.98	73.32	-3	0.555

^*^ = *p* < 0.1, ** = *p* < 0.05, *** = *p* < 0.01

Table 4 displays the ATT of integrated health management within a county medical consortium on SMB and QoL among rural T2D patients using four different PSM modalities. In term of 1:4 nearest neighbor matching, the patient SMB scores in the treatment and control groups were 40.13 and 35.47, respectively, and the ATT was 4.67 (*p*-values < 1%). The ATT for 1:4 intra-caliper nearest neighbor matching, 1:1 nearest neighbor matching, and kernel matching demonstrated significant positive values of 4.67, 4.34, and 4.42, respectively. The results of 1:4 nearest neighbor matching indicate that the QoL scores in the treatment and control groups were 114.99 and 114.10, respectively, and the ATT value was 0.89 (*p*-values < 10 %). The ATT values for the other three matching methods were significant positive values of 0.90, 1.06, and 0.89, respectively. This suggests that after controlling for confounding bias (caused by observable variables), the implementation of integrated health management within a county medical consortium significantly improved rural T2D patients’ SMB and QoL.

**Table 4:** The ATT of integrated health management within a county medical consortium on SM and QoL

Modalities	SM				QoL			
	Treated	Untreated	ATT	t	Treated	Untreated	ATT	t
Unmatched	40.13	35.86	4.27	11.91***	115.32	111.49	3.82	9.26***
1:4 Nearest neighbor matching	40.13	35.47	4.67	11.23***	114.99	114.10	0.89	1.81*
1:4 Intra-caliper nearest neighbor matching	40.13	35.46	4.67	11.24***	114.99	114.10	0.90	1.81*
1:1 Nearest neighbor matching	40.13	35.79	4.34	9.08***	114.99	113.93	1.06	1.81*
Kernel matching	40.13	35.71	4.43	11.40***	114.99	114.10	0.89	1.95*

^*^ = *p* < 0.1, ** = *p* < 0.05, *** = *p* < 0.01

### Quantile regression results

The 1:4 nearest neighbor matching maximally expands the pool of potential matches. This increases the chances of finding suitable matches and improves the balance between treatment and control groups. Therefore, we employed the sample frequencies derived from the 1:4 nearest neighbor matching as weighting factors to conduct the frequency-weighted quantile regression. The results are exhibited in Table 5. Implementation of integrated health management within a county medical consortium was significantly associated with higher SMB in rural T2D patients (coef = 4.15). Self-efficacy and support from the physician/healthcare team were negatively correlated with SMB (coef = -0.65, coef = -3.65). However, SMB correlated significantly and positively with support from family or friends and support from the neighborhood/community (coef = 1.21, coef = 3.20). Diabetes-related distress and diabetes-related knowledge both demonstrated significant and positive association with SMB (coef = 0.19, coef = 0.07). SMB correlated significantly and positively with Co-morbidity (coef = 0.88). Additionally, demographic factors including age, gender, marital status, and educational level demonstrated significant association with SMB.

**Table 5:** Frequency-weighted quantile regression on SM and QoL in the matched sample

Variable	SM			QoL		
	coef.	Std. Err.	95% CI	coef.	Std. Err.	95% CI
Implementation of integrated health management	4.15***	0.24	(3.69, 4.61)	1.54***	0.27	(1.01, 2.06)
Self-efficacy	-0.65***	0.10	(-0.84, -0.47)	1.64***	0.11	(1.43, 1.86)
Support from physician/health care team	-3.65***	0.19	(-4.03, -3.27)	0.10	0.22	(-0.34, 0.53)
Support from family and friends	1.21***	0.16	(0.89, 1.52)	-2.4***	0.18	(-2.78, -2.07)
Support from neighborhood/community	3.20***	0.11	(2.98, 3.42)	0.45***	0.13	(0.20, 0.70)
Diabetes-related distress	0.19***	0.02	(0.16, 0.22)	-0.35***	0.02	(-0.38, -0.32)
Diabetes-related knowledge	0.07***	0.01	(0.05, 0.08)	0.03***	0.01	(0.01, 0.04)
Gender: female (ref: male)	-0.53**	0.21	(-0.95, -0.12)	-0.86***	0.24	(-1.33, -0.39)
Age (year) ≥65 (ref: <65)	-0.59***	0.22	(-1.02, -0.16)	-1.80***	0.25	(-2.29, -1.31)
Personal income ($) (ref: ≤149						
>149 and ≤297	0.89**	0.37	(0.16, 1.62)	0.07	0.42	(-0.76, 0.90)
>297 and ≤743	-0.06	0.41	(-0.86, 0.74)	-4.42***	0.47	(-5.33, -3.50)
>743	1.19***	0.41	(0.38 2.00)	0.49	0.47	(-0.43, 1.41)
Education (ref: elementary school and below)						
Junior high school	1.44***	0.27	(0.93, 1.96)	2.22***	0.30	(1.63, 2.81)
High school and above	1.19***	0.38	(0.44, 1.94)	1.01**	0.44	(0.16, 1.87)
Marital status (ref: single, widowed, divorced, and other)	0.76***	0.25	(0.26, 1.25)	1.96***	0.29	(1.40, 2.51)
Employment status (ref: unemployed)						
Employed	-0.03	0.24	(-0.49, 0.44)	-3.21***	0.27	(-3.74, -2.68)
Retired	-0.89	0.75	(-2.36, 0.59)	-2.86***	0.85	(-4.53, -1.19)
Co-morbidity (ref: diabetes only)	0.88***	0.20	(0.48, 1.27)	-0.67***	0.23	(-1.12, -0.22)
Incapacity (ref: without incapacity)	1.27***	0.38	(0.52, 2.02)	-3.62***	0.43	(-2.77, -4.47)
Self-reported health	-0.02**	0.01	(-0.04, -0.01)	-0.00	0.01	(-0.02, 0.02)
_cons	35.76***	1.25	(33.31, 38.21)	115.95***	1.42	(113.16, 118.73)
Observations	4117			4117		

*CI* Credibility interval

^*^ = *p* < 0.1, ** = *p* < 0.05, *** = *p* < 0.01

The findings from QoL regression revealed a significant and positive correlation between the implementation of integrated health management and QoL of rural T2D patients (coef = 1.54). self-efficacy and support from the neighborhood/community were significantly and positively associated with patients’ QoL (coef = 1.64, coef = 0.45), whereas support from family and friends demonstrated a significantly negative association (coef = -2.43). Diabetes-related distress and QoL were significantly negatively correlated (coef = -0.35); however, the diabetes-related knowledge demonstrated significantly positive correlation with QoL (coef = 0.03). QoL correlated significantly and positively with Co-morbidity (coef = -0.67). Demographic factors such as age, gender, marital status, and educational level were also significantly associated with QoL.

Table 6 demonstrates the association between follow-up service for integrated health management and both SMB and QoL among patients in the treatment group. The results showed a significant and positive correlation between the frequency of follow-up service and SMB in T2D patients (coef = 2.73). SMB was significantly poorer in patients who received on-call follow-up services (coef = -3.79). Patients treated for a condition other than diabetes demonstrated relatively poorer SMB (coef = -2.89), in addition to those who received medication guidance (coef = -5.24). However, patients who received behavioral guidance showed better SMB (coef = 1.50). Based on the results of QoL regression, T2D patients who received follow-up services more than twice in the previous three months had better QoL (coef = 1.79). Patients who received follow-up services at medical institutions had lower QoL (coef = -2.01). However, health behavior guidance showed significant positive correlation with patients’ QoL (coef = 2.55); additionally, patients treated for a condition other than diabetes had a relatively better QoL (coef = 2.32).

**Table 6:** The association between follow-up service for integrated health management and SM/QoL in the treatment group

Variable	SM			QoL		
	coef.	Std. Err.	95% CI	coef.	Std. Err.	95% CI
Follow-up management frequency > 2 times (ref:≤2 times)	2.73***	0.77	(1.23, 4.24)	1.79**	0.85	(0.13, 3.45)
Follow-up management mode						
Households and home visiting	-1.99	2.90	(-7.68, 3.69)	5.00	3.20	(-1.27, 11.28)
Going to a medical institution	0.82	0.63	(-5.02, -2.55)	-2.01**	0.69	(-0.45, 2.27)
On call	-3.79***	0.81	(-0.77, 2.42)	0.91	0.90	(-3.77, -0.25)
Follow-up management content						
Measuring blood glucose	-4.58	3.21	(-10.87, 1.72)	0.93	3.54	(-6.02, 7.87)
Inquiring about diabetes symptoms	-0.62	0.96	(-2.50, 1.27)	0.43	1.06	(-1.65, 2.50)
Medication guidance	-5.24***	0.80	(-6.81, -3.66)	1.22	0.89	(-0.51, 2.96)
Treatment for a condition other than diabetes	-2.89***	0.64	(-4.15, -1.62)	2.32***	0.71	(0.93, 3.72)
Health behavior guidance	1.50**	0.62	(0.29, 2.72)	2.55***	0.68	(1.21, 3.89)
_cons	64.84***	5.64	(53.77, 75.91)	122.28***	6.23	(110.06, 134.50)
Observations	887			887		

*CI* Credibility interval

^*^ = *p* < 0.05, ** = *p* < 0.01, *** = *p* < 0.001

## Discussion

Currently, individuals diagnosed with diabetes in numerous countries typically attain scores below 50% of the maximum possible on scales evaluating SMB and QoL [5–8]. The level of self-management and QoL among individuals with T2D is suboptimal in both, China and worldwide. This holds especially true for rural areas of China, where patients have lower health literacy, poorer living environments, or lower quality of healthcare and consequently have lower levels of self-management and QoL. Integrated health management within a county medical consortium provides a promising model for improving SMB and QoL among rural T2D patients. In this study, we initially employed PSM to precisely estimate the ATT of such management approaches on SMB and QoL. Then, quantile regression was also employed to estimate the relationship between the implementation of integrated health management, sociodemographic factors, follow-up services (offered during integrated health management) and both SMB and QoL in rural T2D patients.

The results of this study showed the median score of patient SMB to be 38, which is lower than the values reported by prior studies on urban patients [31]; this indicates that SMB is inferior among rural T2D patients. In particular, blood glucose monitoring and foot care were found to be the most suboptimal dimensions in this study. Patients had a median QoL value of 114 and the physiology dimension had the lowest score; this indicated that bodily functions were severely affected by diabetes.

PSM is a suitable nonparametric method for estimating the net effect of policy implementation. Our study revealed the ATT values of integrated health management within a county medical consortium on SMB and QoL in rural T2D patients to be significantly positive; they ranged from 4.34 to 4.67 and 0.89 to 1.06, respectively, based on the four different PSM modalities employed. The results of weighted quantile regression also confirmed the positive correlation of the implementation of integrated health management with SMB and QoL in these patients. Therefore, the findings substantiated that integrated health management within a county medical consortium may effectively improve SMB and QoL in rural T2D patients. It may overcome fragmentation of traditional diabetic health education models by integrating healthcare resources within a county area, thereby delivering continuous services (including but not limited to blood glucose monitoring, diabetes symptom counseling, medication guidance, and health behavior guidance) [14]; these interventions may enhance patient SMB and improve their QoL. Therefore, policy initiatives should continue to increase the establishment of county-level medical consortiums in rural China, with particular emphasis on refinement of integrated health management strategies for individuals with chronic conditions. The findings also provide valuable insights for the advancement of chronic disease management in rural areas of other developing countries.

The health management team within the county medical consortium is primarily responsible for providing follow-up services of integrated health management. The findings of quantile regression in the treatment group showed that SMB and QoL of rural T2D patients were significantly influenced by the frequency, mode, and content of follow-up services. The higher the frequency of follow-up, the better were the levels of SMB and QoL; these findings are similar to those of previous studies [32]. Patients who received on-call follow-up services had poorer SMB; this implied that this mode was relatively less effective in enhancing SMB when compared to other follow-up service modes. According to a recent study, the impact of telephone-based health management on improving SMB in individuals with diabetes may depend on factors such as call duration and content [33]. Furthermore, there is a growing body of literature that supports the integration of ancillary services, such as health coaching, in conjunction with remote monitoring technologies for diabetes management. These studies suggest that such interventions may have additive and synergistic effects on patient outcomes. Given these findings, the adoption of remote monitoring technology could be considered a viable way for addressing existing gaps in the management of this population [34, 35]. Further research is necessary to explore the reasons behind the observed differences and identify more effective modes of follow-up services for rural T2D patients. The QoL of T2D patients who visit medical institutions to receive follow-up services was worse. The consideration to be made is that patients who present to a medical institution may have poor underlying health conditions, explaining the reported low QoL. The SMB of T2D patients who received medication guidance was relatively poor, probably because patients may receive instructions to prioritize diabetes medication over other dimensions of SMB. This may lead to significant neglect of other aspects of SMB. Follow-up services should therefore aim to guide these patients to focus on all aspects of self-management, rather than emphasizing on a single component. Rural T2D patients who received additional treatment for conditions other than diabetes demonstrated poorer SMB but better QoL. This suggests that although the treatment and management of other diseases may hamper SMB in diabetes, it improves QoL in these patients. Similar to findings from earlier research [36], both SMB and QoL in rural T2D patients improved with guidance on healthy behaviors.

Among individual factors, self-efficacy demonstrated negative correlation with SMB in rural T2D patients; it may be attributed to the limited educational level of the rural patients (most had only elementary school education or below in our study) and the lack of diabetes-related knowledge (the accuracy rate is only 52.08% in our study). This could have caused a false sense of confidence and overestimation of the ability to self-manage diabetes, ultimately resulting in misjudgment regarding appropriate SMB. A study also showed that self-efficacy of overconfident people negatively correlates with their performance [37]. Consistent with earlier studies [38], this study found that diabetes-related knowledge is beneficial for patient SMB and QoL. In addition, our study revealed a positive correlation between diabetes-related distress and patient SMB, contrary to previous studies [39]. Experiencing symptoms of distress often motivates individuals to manage their condition, leading to a greater focus on SMB. Additionally, diabetes-related distress may act as a reminder to adhere to treatment plans and maintain healthy lifestyle [32]. However, similar to findings from previous studies, diabetes-related distress was negatively associated with patient QoL in this study [40]. Support from healthcare professionals was negatively associated with patient SMB; this may be due to T2D patients with poorer SMB require more support from healthcare professionals. Support from family and friends had a positive association with patient SMB. Family members and friends may crucially monitor healthy behaviors and provide significant emotional and financial support. However, patients receiving more support from friends and family reported lower QoL; this may be due to the fact that individuals with poor health in rural areas tend to receive more support from family and friends. Additionally, rural T2D Patients receiving support from the community reported better SMB and QoL. Some patients with multiple diseases exhibit better SMB and poorer QoL. The underlying reasons may involve various factors. These could include the increased awareness and necessity for self-management due to managing multiple health conditions, the motivation to maintain control over their health, and the adoption of coping strategies to manage complex treatment regimens among patients with multiple diseases. However, despite their efforts in self-management, the cumulative burden of managing multiple diseases may contribute to physical and emotional strain, leading to a diminished overall QoL.

### Policy implications

This study underscores key policy implications crucial for enhancing integrated health management within medical communities, particularly for patients with chronic diseases: (1) Enhancement of governmental accountability. It is critical to strengthen government responsibility by adopting multiple measures to refine the integrated health management models. This entails boosting stakeholder motivation, clarifying rights and responsibilities, and reinforcing supervision and accountability mechanisms to ensure smooth implementation and effectiveness within the medical community. (2) Diversification of follow-up health services. This involves not only enhancing the content of follow-up services but also adjusting the methods and programs based on actual service dynamics and patient feedback to ensure responsiveness and adaptability. (3) Intensification of health education and behavioral guidance within the integrated health management. Efforts should focus on correcting health misconceptions, reshaping patient attitudes, promoting active engagement in disease management, and thereby enhancing SMB and QoL. (4) Leveraging social support to promote behavioral management. Utilizing emotional support from family and friends, as well as advocating for community support in resource allocation and optimizing environmental facilities, are recommended strategies for cultivating conducive conditions for the SMB.

### Limitation and strengths

To the best of our knowledge, prior research has not examined the influence of integrated health management within a county medical consortium on SMB and QoL among rural Chinese patients with T2D. The study corroborates the effectiveness of such management approaches in improving SMB and QoL among this group. The findings provide valuable insights for the advancement of chronic disease management in rural areas of other developing countries. Our study had certain limitations. First, despite using PSM to estimate the ATT, confounding bias caused by unobservable variables could not be completely eliminated. Second, as the information was collected through an interview process involving standardized questions, scales, and self-reported responses, the presence of recall bias was inevitable. Third, as the study included rural individuals with T2D from a particular pilot region in Jiangsu, generalization of the findings to other regions or populations may be limited.

## Conclusion

Our findings suggest that integrated health management within a county medical consortium can effectively improve SMB and QoL in rural T2D patients. The frequency, mode, and content of follow-up services (offered during integrated health management) were significantly associated with SMB and QoL. The findings offer strong empirical evidence supporting the importance of promoting integrated health management within a medical consortium as a key priority in the ongoing healthcare system reform in China. This study also offers invaluable insights for the advancement of chronic disease management in rural areas of other developing countries. Policy initiatives should therefore continue to increase the establishment of county-level medical consortiums in rural China, with particular emphasis on the refinement of integrated health management strategies for individuals with chronic conditions. Additionally, social support, self-efficacy, diabetes-related knowledge, and other sociodemographic factors were found to significantly correlate with patient SMB and QoL. Healthcare providers should therefore consider these factors while formulating interventions and management plans for rural individuals with T2D. Future research should segment diabetic patients into homogeneous groups to investigate the relationship between integrated health management and patients' SMB and QoL. This facilitates targeted allocation of integrated health management resources to prioritize patients at higher risk.

### Supplementary Information

Additional file 1: Table S1. Covariate imbalance testing of 1:4 intra-caliper nearest neighbor matching. Table S2. Covariate imbalance testing of 1:1 nearest neighbor matching. Table S3. Covariate imbalance testing of kernel matching. Figure S1. Standardized percentage bias across covariates of 1:4 intra-caliper nearest neighbor matching. Figure S2. Standardized percentage bias across covariates of 1:1 nearest neighbor matching. Figure S3. Standardized percentage bias across covariates of kernel matching.

Additional file 2. The Survey Scale Used in This Study.

## Data Availability

The datasets used and/or analyzed during the current study are available from the corresponding author on reasonable request.

## Abbreviations

T2D: Type 2 diabetes
SMB: Self-management behavior
QoL: Quality of life
PSM: Propensity score matching
ATT: Average treatment effect on treated
WHO: The World Health Organization
ICCC: Innovative Care for Chronic Conditions
CDSM: Chronic Disease Self-Management
D-QoL: The Diabetes-specific Quality-of-Life
SDSCA: The Summary of Diabetes Self-Care Activities
SECD6: The Self-Efficacy for Managing Chronic Disease 6-Item
CIRS: The Chronic Illness Resources Survey
AD-knowl: The Audit of Diabetes Knowledge
PAID: The Problem Areas in Diabetes Scale
EQ-VAS: The EuroQol Visual Analogue Scale
IQR: Interquartile range
CI: Credibility intervals
