# Supplementary material for: Does integrated health management within a county medical consortium improve rural type 2 diabetic patients’ self-management behavior and quality of life? An empirical analysis from Eastern China
Source: BMC Public Health. 2024 May 29;24:1439. doi: 10.1186/s12889-024-18885-0 (PMC11138014; doi:10.1186/s12889-024-18885-0)
Supplement: Supplementary file 1 — Additional file 1: Table S1. Covariate imbalance testing of 1:4 intra-caliper nearest neighbor matching. Table S2. Covariate imbalance testing of 1:1 nearest neighbor matching. Table S3. Covariate imbalance testing of kernel matching. Figure S1. Standardized percentage bias across covariates of 1:4 intra-caliper nearest neighbor matching. Figure S2. Standardized percentage bias across covariates of 1:1 nearest neighbor matching. Figure S3. Standardized percentage bias across covariates of kernel matching. [file 12889_2024_18885_MOESM1_ESM.docx]

**Table S1 Covariate imbalance testing (1:4 Intra-caliper nearest neighbor matching)**

| Variable | 1:4 Intra-caliper nearest neighbor matching | | | |
| --- | --- | --- | --- | --- |
|  | Treated | Untreated | %Bias | *P* value |
| Self-efficacy | 6.592 | 6.592 | 0 | 0.993 |
| Support from physician/health care team | 4.224 | 4.231 | -1.2 | 0.801 |
| Support from family and friends | 3.723 | 3.721 | 0.4 | 0.931 |
| Support from neighborhood/community | 3.031 | 3.035 | -0.5 | 0.916 |
| Diabetes-related distress | 10.752 | 11.300 | -7.2 | 0.126 |
| Diabetes-related knowledge | 51.015 | 50.331 | 4.2 | 0.333 |
| Gender | 1.625 | 1.627 | -0.4 | 0.94 |
| Age | 67.473 | 67.869 | -4.7 | 0.326 |
| Personal income | 2.571 | 2.571 | 0.1 | 0.984 |
| Education | 1.321 | 1.321 | 0 | 0.992 |
| Marital status | 0.830 | 0.830 | -0.2 | 0.974 |
| Employment status | 1.377 | 1.382 | -0.9 | 0.854 |
| Co-morbidity | 0.673 | 0.693 | -4.3 | 0.373 |
| Incapacity | 0.076 | 0.088 | -4.3 | 0.363 |
| Self-reported health | 72.980 | 73.319 | -3 | 0.558 |

**Figure S1 Standardized percentage bias across covariates (1:4 Intra-caliper nearest neighbor matching)**

**Table S2 Covariate imbalance testing (1:1 Nearest neighbor matching)**

| Variable | 1:1 Nearest neighbor matching | | | |
| --- | --- | --- | --- | --- |
|  | Treated | Untreated | %Bias | *P* value |
| Self-efficacy | 6.592 | 6.603 | -0.9 | 0.836 |
| Support from physician/health care team | 4.224 | 4.224 | 0.1 | 0.981 |
| Support from family and friends | 3.723 | 3.738 | -2.2 | 0.647 |
| Support from neighborhood/community | 3.031 | 3.076 | -4.9 | 0.295 |
| Diabetes-related distress | 10.752 | 11.230 | -6.3 | 0.178 |
| Diabetes-related knowledge | 51.015 | 50.766 | 1.5 | 0.719 |
| Gender | 1.625 | 1.632 | -1.5 | 0.762 |
| Age | 67.473 | 68.052 | -6.9 | 0.143 |
| Personal income | 2.571 | 2.619 | -5.1 | 0.303 |
| Education | 1.321 | 1.338 | -2.7 | 0.585 |
| Marital status | 0.830 | 0.833 | -1 | 0.845 |
| Employment status | 1.377 | 1.419 | -7.7 | 0.11 |
| Co-morbidity | 0.673 | 0.679 | -1.3 | 0.795 |
| Incapacity | 0.076 | 0.077 | -0.4 | 0.927 |
| Self-reported health | 72.980 | 73.263 | -2.5 | 0.623 |

**Figure S2 Standardized percentage bias across covariates (1:1 nearest neighbor matching)**

**Table S3 Covariate imbalance testing (Kernel matching)**

| Variable | Kernel matching | | | |
| --- | --- | --- | --- | --- |
|  | Treated | Untreated | %Bias | *P* value |
| Self-efficacy | 6.592 | 6.566 | 2.1 | 0.653 |
| Support from physician/health care team | 4.224 | 4.223 | 0.2 | 0.963 |
| Support from family and friends | 3.723 | 3.714 | 1.5 | 0.755 |
| Support from neighborhood/community | 3.031 | 3.034 | -0.4 | 0.94 |
| Diabetes-related distress | 10.752 | 11.244 | -6.5 | 0.173 |
| Diabetes-related knowledge | 51.015 | 50.605 | 2.5 | 0.562 |
| Gender | 1.625 | 1.631 | -1.2 | 0.804 |
| Age | 67.473 | 67.647 | -2.1 | 0.67 |
| Personal income | 2.571 | 2.565 | 0.7 | 0.88 |
| Education | 1.321 | 1.324 | -0.4 | 0.94 |
| Marital status | 0.830 | 0.838 | -2.2 | 0.647 |
| Employment status | 1.377 | 1.376 | 0.2 | 0.963 |
| Co-morbidity | 0.673 | 0.691 | -3.9 | 0.415 |
| Incapacity | 0.076 | 0.085 | -3 | 0.527 |
| Self-reported health | 72.980 | 72.833 | 1.3 | 0.803 |

**Figure S3 Standardized percentage bias across covariates (Kernel matching)**
