# Supplementary material for: Does integrated health management within a county medical consortium improve rural type 2 diabetic patients’ self-management behavior and quality of life? An empirical analysis from Eastern China
Source: BMC Public Health. 2024 May 29;24:1439. doi: 10.1186/s12889-024-18885-0 (PMC11138014; doi:10.1186/s12889-024-18885-0)
Supplement: Supplementary file 2 — Additional file 2. The Survey Scale Used in This Study. [file 12889_2024_18885_MOESM2_ESM.docx]

**Demonstrate of the Relevant Scales**

**Appendix A**

**The Self-efficacy for Managing Chronic Disease 6-Item Scale (SECD6)**

We would like to know how confident you are in carrying out certain activities, so please tick the numbers that correspond to how confident you have been in dealing with these matters recently. "1" means "not at all confident" and "10" means "absolutely confident".

| 1. How confident are you that you can manage the fatigue from your illness so that it doesn't interfere with the things you want to do? |
| --- |
| not at all confident 1 2 3 4 5 6 7 8 9 10 absolutely confident |
| 2. How confident are you that you can manage the physical discomforts of your illness so that they don't interfere with what you want to do? |
| not at all confident 1 2 3 4 5 6 7 8 9 10 absolutely confident |
| 3. How confident are you that you can manage the low mood brought on by your illness so that it doesn't interfere with what you want to do? |
| not at all confident 1 2 3 4 5 6 7 8 9 10 absolutely confident |
| 4. How confident are you that you can manage the symptoms or health problems associated with your illness so that they don't get in the way of what you want to do? |
| not at all confident 1 2 3 4 5 6 7 8 9 10 absolutely confident |
| 5. How confident are you in taking steps to improve your health in order to reduce the need to see a doctor? |
| not at all confident 1 2 3 4 5 6 7 8 9 10 absolutely confident |
| 6. How confident are you in doing other things, not just taking medication, to lessen the impact of your illness on your daily life? |
| not at all confident 1 2 3 4 5 6 7 8 9 10 absolutely confident |

**Appendix B**

**The Chronic Illness Resource Survey Scale (CIRS)**

We would like to know about the various resources you use for disease management, please tick the appropriate box based on how far you have chosen based on your experience in the last 6 months, depending on your circumstances.

| Items | Please tick the appropriate box | | | | |
| --- | --- | --- | --- | --- | --- |
|  | Never | Occa-  sionally | Mode-  rately | More often | Very  often |
| **Doctors and nurses** | | | | | |
| 1. Whether your doctor has discussed with you things related to the treatment of your disease? (e.g., exercise, diet, medication, regular office visits, etc.) | □ | □ | □ | □ | □ |
| 2. Whether your doctor or nurse listened carefully to your description of your condition? | □ | □ | □ | □ | □ |
| 3. Whether your doctor or nurse told you the results of your laboratory tests (e.g. blood pressure, blood sugar, glycated haemoglobin, cholesterol, etc.) in a way that you can understand them? | □ | □ | □ | □ | □ |
| 4. Importance of healthcare professionals in the disease management process. | □ | □ | □ | □ | □ |
| **Family and friends** | | | | | |
| 5. Whether your family or friends exercised with you? | □ | □ | □ | □ | □ |
| 6. Whether your family or friends reminded you to take your medication? | □ | □ | □ | □ | □ |
| 7. Did they choose or ask for healthy food choices (low fat and low sugar) when you are with family or friends? | □ | □ | □ | □ | □ |
| 8. Whether your family or friends listened carefully to your description of your condition? | □ | □ | □ | □ | □ |
| 9. The importance of support from your family or friends in managing the disease | □ | □ | □ | □ | □ |
| **Neighbourhoods and communities** | | | | | |
| 10. Did you walk or do outdoor exercise near your home? | □ | □ | □ | □ | □ |
| 11. Did you walk or take other exercise with your neighbours? | □ | □ | □ | □ | □ |
| 12. Importance of neighbourhoods and communities in the disease management process | □ | □ | □ | □ | □ |

**Appendix C**

**The Scale of Diabetes Self-Care Activities (SDSCA)**

We would like to know how well you have self-managed in the last seven days, please tick the number of days you have met.

| Items | Please tick the appropriate box | | | | | | |
| --- | --- | --- | --- | --- | --- | --- | --- |
|  | 1  day | 2  days | 3  days | 4  days | 5  days | 6  days | 7  days |
| 1. How many days in the past 7 days did you follow a diabetic diet? | □ | □ | □ | □ | □ | □ | □ |
| 2. In the past 1 month, on average, how many days per week did you follow a diabetic diet? | □ | □ | □ | □ | □ | □ | □ |
| 3. In the past 7 days, how many days did you consume more than 5 types of fruit and vegetables? | □ | □ | □ | □ | □ | □ | □ |
| 4. In the past 7 days, on how many days did you consume foods high in fat, such as beef, lamb, or full-fat dairy products? | □ | □ | □ | □ | □ | □ | □ |
| 5. In the past 7 days, how many days did you exercise for more than 30 minutes (30 minutes is the duration of continuous activity, including walking)? | □ | □ | □ | □ | □ | □ | □ |
| 6. In addition to doing chores or work, how many days in the past 7 days did you participate in some special exercise programme (e.g. swimming, walking, jogging or cycling)? | □ | □ | □ | □ | □ | □ | □ |
| 7. How many days in the past 7 days have you measured your blood sugar? | □ | □ | □ | □ | □ | □ | □ |
| 8. How many days in the past 7 days have you monitored your blood glucose according to the number of blood glucose measurements recommended by your healthcare provider? | □ | □ | □ | □ | □ | □ | □ |
| 9. How many days in the past 7 days have you checked your feet (for any abnormalities such as tears, ulcers, blackening, etc.)? | □ | □ | □ | □ | □ | □ | □ |
| 10. How many days in the past 7 days have you inspected the inside of your shoes (for foreign objects, flatness, comfort)? | □ | □ | □ | □ | □ | □ | □ |
| 11. In the past 7 days, how many days did you take your medication or insulin injections correctly as required by your doctor? | □ | □ | □ | □ | □ | □ | □ |

**Appendix D**

**The Problem Areas in Diabetes Scale (PAID)**

We would like to know how much diabetes interferes with your daily life, please tick the number that matches based on the last 3 months.

| Items | Please tick the appropriate box | | | | |
| --- | --- | --- | --- | --- | --- |
|  | Not a problem | Very small problem | Moderate problem | Somewhat serious problem | Serious problem |
| 1. Did you have clear and specific goals for your diabetes care? | □ | □ | □ | □ | □ |
| 2. Did you find it difficult to reach your diabetes treatment plan? | □ | □ | □ | □ | □ |
| 3. Did you feel scared when you think about your diabetes? | □ | □ | □ | □ | □ |
| 4. Has diabetes had a negative impact on your social life? | □ | □ | □ | □ | □ |
| 5. Did you feel deprived of the right to enjoy food and meals freely? | □ | □ | □ | □ | □ |
| 6. Did you feel depressed when you think about having diabetes? | □ | □ | □ | □ | □ |
| 7. Have your emotions and feelings changed as a result of having diabetes? | □ | □ | □ | □ | □ |
| 8. Did you feel beaten down by diabetes? | □ | □ | □ | □ | □ |
| 9. Did you worry about blood sugar reactions? | □ | □ | □ | □ | □ |
| 10. Did you feel angry when you think about having diabetes? | □ | □ | □ | □ | □ |
| 11. Did you worry about food and eating all the time? | □ | □ | □ | □ | □ |
| 1. Did you concern about the high probability of serious complications in the future? | □ | □ | □ | □ | □ |
| 13. Feeling guilty or anxious when you do not comply with the management of your diabetes? | □ | □ | □ | □ | □ |
| 14. Feeling unacceptable that you have diabetes? | □ | □ | □ | □ | □ |
| 15. Did you dissatisfy with your diabetes doctor? | □ | □ | □ | □ | □ |
| 16. Did you feel that diabetes takes too much of your mental and physical energy every day? | □ | □ | □ | □ | □ |
| 17. Did you feel lonely because of diabetes? | □ | □ | □ | □ | □ |
| 18. Did you feel that friends and family do not support your efforts to manage your diabetes? | □ | □ | □ | □ | □ |
| 19. Managing the complications of diabetes? | □ | □ | □ | □ | □ |
| 20. Did you feel "exhausted" by the constant effort required to manage diabetes? | □ | □ | □ | □ | □ |

**Appendix E**

**The Audit of Diabetes Knowledge Scale (ADKnowl)**

We would like to know how much you know about diabetes and the indicators that can reflect your diabetes control status. Please answer the following questions as honestly and clearly as possible, make judgements according to your own understanding, and mark "√" in the "True" or "False" box. "If you do not understand the question or do not know the correct answer, please tick the "Don't know" box.

| Items | Please tick the appropriate box | | |
| --- | --- | --- | --- |
|  | True | False | Don’t know |
| 1. The following statements about diabetes, do you think that | | | |
| A. Diabetes can be controlled with treatment | □ | □ | □ |
| B. The presence of small amounts of glucose in the urine is normal | □ | □ | □ |
| C. Diabetes may heal itself after a while | □ | □ | □ |
| D. Emotional stress may affect blood sugar levels | □ | □ | □ |
| E. Blood glucose levels do not affect the risk of developing diabetic complications | □ | □ | □ |
| 2. When applying oral medication for the treatment of diabetes, you believe that oral medication: (This option is for diabetic patients treated with oral medication) | | | |
| A. Helps lower blood sugar levels | □ | □ | □ |
| B. Does not need to be taken daily | □ | □ | □ |
| C. If no glucose is detected in the urine then the drug can be stopped | □ | □ | □ |
| D. sometimes leads to hypoglycaemia | □ | □ | □ |
| 3. If you are treated with oral hypoglycaemic drugs, perform the following activities when you are sick and have lost your appetite and have not eaten: (This option is for people with diabetes who are treated with oral medication) | | | |
| A. Testing blood sugar | □ | □ | □ |
| B. Continue to take medication | □ | □ | □ |
| C. stop taking the medication if the blood glucose level is too low | □ | □ | □ |
| D. If you are so thirsty that you have to drink a lot of water, you need to see a doctor | □ | □ | □ |
| 4. If you are on insulin therapy, perform the following activities when you are sick and experience loss of appetite without eating: (This option is for insulin-treated diabetics) | | | |
| A. Reduced insulin dosage | □ | □ | □ |
| B. Frequent monitoring of blood glucose and urine ketone levels | □ | □ | □ |
| C. add additional rapid-acting insulin if urine ketones are positive | □ | □ | □ |
| D. If you are so thirsty that you have to drink a lot of water, you need to see a doctor | □ | □ | □ |
| 5. A description of hypoglycaemia: | | | |
| A. very little glucose in the plasma | □ | □ | □ |
| B. More glucose in the plasma | □ | □ | □ |
| C. Increased physical activity can cause hypoglycaemia | □ | □ | □ |
| D. Alcohol can cause hypoglycaemia | □ | □ | □ |
| E. Too much insulin can cause hypoglycaemia | □ | □ | □ |
| 6. Which of the following are symptoms of hypoglycaemia: | | | |
| A. Slurred speech | □ | □ | □ |
| B. Thirst | □ | □ | □ |
| C. Sweating | □ | □ | □ |
| D. Vertigo | □ | □ | □ |
| E. Confusion | □ | □ | □ |
| F. Polyuria | □ | □ | □ |
| 7. If you experience hypoglycaemia: | | | |
| A. Drink sugary drinks immediately | □ | □ | □ |
| B. Eat a piece of chocolate or a biscuit immediately | □ | □ | □ |
| C. rest for 15 minutes after managing hypoglycaemia | □ | □ | □ |
| 8. The role of sport: | | | |
| A. Lowering blood sugar levels | □ | □ | □ |
| B. Elevate blood sugar levels | □ | □ | □ |
| C. Increase glucose levels in the urine | □ | □ | □ |
| D. Does not change blood glucose levels | □ | □ | □ |
| 9. If you are on insulin therapy, 1 hour before exercise, perform the following activities: (This option is for insulin-treated diabetics) | | | |
| A. Blood glucose measurement | □ | □ | □ |
| B. eating the same amount of food reduces insulin dosage | □ | □ | □ |
| C. Eat more food than usual if insulin dosage is unchanged | □ | □ | □ |
| D. Reduce the amount of food and increase the amount of insulin used | □ | □ | □ |
| E. If hypoglycaemia occurs, speed up the consumption of sugary foods | □ | □ | □ |
| 10. If you are treated with insulin, perform the following activities: (This option is for insulin-treated diabetics) | | | |
| A. If you have a chocolate bar between meals, you need to increase your insulin dosage | □ | □ | □ |
| B. If you eat a snack 1 hour before a meal, you can wait until the next meal and do not need to increase your insulin dosage | □ | □ | □ |
| C. Any additional meal requires an increase in insulin dosage | □ | □ | □ |
| 11. The effect of the following foods on blood sugar: | | | |
| A. Sweets can affect blood sugar | □ | □ | □ |
| B. Foods with a lot of starch (e.g. potatoes, bread, etc.) affect blood sugar levels | □ | □ | □ |
| C. Foods high in protein (e.g. meat, cheese, etc.) affect blood sugar levels | □ | □ | □ |
| D. whole foods have a higher effect on blood sugar than low-fat foods | □ | □ | □ |
| E. 75 grams of glucose requires more insulin than 2 tael buns containing 75 grams of glucose | □ | □ | □ |
| F. Grapefruit and others have less effect on blood sugar, so you can eat them as much as you like | □ | □ | □ |
| G. Fresh, unsweetened fruit juices can be consumed freely | □ | □ | □ |
| 12. Descriptions of the food in question: | | | |
| A. People with diabetes should avoid eating any food containing sugar | □ | □ | □ |
| B. Protein-containing foods can be eaten in large quantities | □ | □ | □ |
| C. Fried foods are usually high in fat | □ | □ | □ |
| D. Pastries and cakes are high in fat | □ | □ | □ |
| E. See-through foods such as melon seeds and peanuts can be eaten in unlimited quantities. | □ | □ | □ |
| F. Sugar-free foods (such as sugar-free mooncakes or sugar-free snacks) can be eaten more often | □ | □ | □ |
| G. Limiting salt intake lowers blood pressure | □ | □ | □ |
| H. High-fat foods increase the risk of chronic diabetes complications | □ | □ | □ |
| I. Foods specially formulated for people with diabetes can be eaten regardless of weight goal | □ | □ | □ |
| 13. Alcohol products usually: | | | |
| A. will lower blood sugar levels after a few hours | □ | □ | □ |
| B. will initially cause a rise in blood sugar | □ | □ | □ |
| C. No calories | □ | □ | □ |
| 14. If you are treated with insulin, perform the following activities: (This option is for insulin-treated diabetics) | | | |
| A. if more beer is consumed, the amount of insulin needs to be increased to control blood sugar | □ | □ | □ |
| B. If you drink 1 glass of white wine (beer) with a meal, you do not need to adjust your insulin dosage | □ | □ | □ |
| C. If 6 or more units of alcohol are consumed in the evening, insulin dosage needs to be reduced to avoid nocturnal hypoglycaemia. (Note: 1 unit of alcohol = ½ bottle of beer, 1 small glass of white wine) | □ | □ | □ |
| 15. Long-term adherence to blood sugar control reduces the risk of damage to which of the following organs: | | | |
| A. Nerve of the foot | □ | □ | □ |
| B. Kidney | □ | □ | □ |
| C. Eyes | □ | □ | □ |
| 16. Periodic inspections include: | | | |
| A. Nerve of the foot | □ | □ | □ |
| B. Blood pressure | □ | □ | □ |
| C. Eyes | □ | □ | □ |
| D. Cholesterol levels | □ | □ | □ |
| E. Examine only the areas where you are having problems | □ | □ | □ |
| 17. Eye examinations using a detector (a special instrument for examining the fundus of the eye): | | | |
| A. This test is needed even if the patient with glucose tolerance has good glycaemic control | □ | □ | □ |
| B. This test is not necessary for diabetics who are on dietary control only | □ | □ | □ |
| C. Screening is recommended because early detection and treatment of fundus disorders can prevent blindness | □ | □ | □ |
| 18. If a diabetic smokes: | | | |
| A. Increased risk of amputation for severe diabetic foot disease | □ | □ | □ |
| B. Increased risk of heart disease | □ | □ | □ |
| C. Increased risk of developing stroke | □ | □ | □ |
| D. does not increase the risk of disease compared to non-diabetics who smoke | □ | □ | □ |
| E. is a good way to control your weight | □ | □ | □ |
| 19. After diabetes, feet need to be checked for warmth, discolouration, infection, calluses (calluses) or injury: | | | |
| A. You or someone else routinely checks once a day | □ | □ | □ |
| B. when wearing new shoes need to be checked | □ | □ | □ |
| C. you need to be checked when you feel unwell | □ | □ | □ |
| D. check only when you develop foot lesions | □ | □ | □ |
| 20. On foot care: | | | |
| A. It's best to choose shoes that are one size larger than your actual shoe size | □ | □ | □ |
| B. Foot soaking is beneficial | □ | □ | □ |
| C. You may have injured your foot but you don't feel it. | □ | □ | □ |
| D. foot injuries will take longer to heal than in non-diabetics | □ | □ | □ |
| E. Wounds are prone to infection if proper care is not taken | □ | □ | □ |
| 21. It is recommended that you build toenails: | | | |
| A. Trimmed to a point | □ | □ | □ |
| B. Trimmed to the tip of the toes (with a rounded shape) | □ | □ | □ |
| 22. Foot problems such as blisters and calluses (calluses) will be safely treated through: | | | |
| A. Specialist podiatrists | □ | □ | □ |
| B. Pedicurist | □ | □ | □ |
| C. Yourself | □ | □ | □ |
| D. Anyone | □ | □ | □ |
| 23. Recommended shoe type for diabetics: | | | |
| A. Shoes with laces | □ | □ | □ |
| B. Soft running shoes | □ | □ | □ |
| C. High heels | □ | □ | □ |
| D. Open-toed shoes | □ | □ | □ |
| E. Pointed Toe Shoes | □ | □ | □ |
| 24. Dry skin on the feet of diabetic patients is recommended: | | | |
| A. Foot rubbing | □ | □ | □ |
| B. Apply moisturiser to feet | □ | □ | □ |
| C. Leave it alone | □ | □ | □ |
| D. See a professional podiatrist | □ | □ | □ |
| 25. On glycosylated haemoglobin: | | | |
| A. can find out if you are experiencing hypoglycaemia | □ | □ | □ |
| B. Reflects the average blood glucose level over the past 8-12 weeks. | □ | □ | □ |
| C. Reflects the average blood glucose level over the past 8-12 days | □ | □ | □ |
| D. Reflects the average blood glucose level over the past 24 hours. | □ | □ | □ |
| 26. The target value for glycated haemoglobin as set out in our diabetes guidelines: | | | |
| A. Maximum value of 6.5 per cent | □ | □ | □ |
| B. Maximum value of 7.0 per cent | □ | □ | □ |

**Appendix F**

**The Diabetes-specific Quality-of-Life (D-QoL)**

Comprehensive evaluation of the health status of diabetic patients (e.g., physical and mental health as well as well-adjusted status in terms of social relations and the surrounding environment); evaluation of the impact of medical measures (e.g., the effectiveness of health education, the effectiveness of medication, etc.) on the patient's health status. Each question in the table asks about the patient's own feelings, so please read the questions and tick the appropriate box. Please read the questions and tick as many as you like. Note that all the questions are only about your feelings in the last two weeks.

| **Physiological dimensions (12 articles)** | | | | |
| --- | --- | --- | --- | --- |
| 1. Overall, how much did diabetes damage your health? | | | | |
| □Not at all | □Some damage | □Moderate | □Very damage | □Extreme damage |
| 1. Did you often have itchy skin, limb numbness, pain and other physical discomfort? | | | | |
| □Not at all | □Occasionally | □About half the time | □Often | □Always |
| 3. How much does physical discomfort interfere with your life? | | | | |
| □Not at all | □Some interference | □Moderate | □Very much interference | □Extreme interference |
| 4. Did you feel that it is becoming increasingly difficult to see? | | | | |
| □Not at all | □Occasionally | □About half the time | □Often | □Always |
| 5. How much did vision loss affect your daily life? | | | | |
| □Not at all | □Somewhat impactful | □Impactful | □Very impactful | □Extremely impactful |
| 6. Did you feel that it is becoming more and more difficult to hear others? | | | | |
| □Not at all | □Occasionally | □About half the time | □Often | □Always |
| 7. How much did hearing loss affect your daily life? | | | | |
| □Not at all | □Somewhat impactful | □Impactful | □Very impactful | □Extremely impactful |
| 8. Did you feel chest pain, chest tightness and palpitations? | | | | |
| □Not at all | □Occasionally | □About half the time | □Often | □Always |
| 9. Did you feel that your skin and feet are easily infected? | | | | |
| □Not at all | □Occasionally | □About half the time | □Often | □Always |
| 10. How much did skin and foot infections affect your life? | | | | |
| □Not at all | □Somewhat impactful | □Impactful | □Very impactful | □Extremely impactful |
| 11. Did you feel less able to react to external things? | | | | |
| □Not at all | □A little decline | □moderate | □A big decline | □An enormous decline |
| 12. Did you always feel hungry? | | | | |
| □Not at all | □Occasionally | □About half the time | □Often | □Always |
| **Psychological dimensions (8 articles)** | | | | |
| 13. Did diabetes often cause problems and inconveniences in your daily life? | | | | |
| □Not at all | □Occasionally | □About half the time | □Often | □Always |
| 14. Did you often think about what diabetes means to you? | | | | |
| □Not at all | □Occasionally | □About half the time | □Often | □Always |
| 15. Were you worried that you will die suddenly? | | | | |
| □Not at all | □Occasionally | □About half the time | □Often | □Always |
| 16. Did diet control bother you? | | | | |
| □Not at all | □Occasionally | □About half the time | □Often | □Always |
| 17. Did it bother you to measure your own urine sugar or check your blood sugar in the hospital on a regular basis? | | | | |
| □Not at all | □Occasionally | □About half the time | □Often | □Always |
| 18. Did you feel nervous or anxious because of your diabetes? | | | | |
| □Not at all | □Occasionally | □About half the time | □Often | □Always |
| 19. Were you satisfied with your current treatment results? | | | | |
| □Extremely satisfied | □Very satisfied | □Satisfied | □Very dissatisfied | □Extremely dissatisfied |
| 1. Did you believe you can beat the disease? | | | | |
| □Not at all | □Somewhat convinced | □Convinced | □Very convinced | □Extremely convinced |
| **Social relations dimension (4 articles)** | | | | |
| 21. In general, has diabetes damaged your relationships? | | | | |
| □Not at all | □Some damage | □moderate | □Very damage | □Extreme damage |
| 22. Did you feel that you are disliked because you have diabetes? | | | | |
| □Not at all | □Occasionally | □About half the time | □Often | □Always |
| 23. Has diabetes had an impact on your status and role in your family or organisation? | | | | |
| □Not at all | □Somewhat impactful | □Impactful | □Very impactful | □Extremely impactful |
| 24. Did you share your experiences, problems and knowledge about diabetes with your neighbours? | | | | |
| □Not at all | □Communicates occasionally | □Communicates | □Communicates often | □Communicates all the time |
| **Therapeutic dimensions (3 articles)** | | | | |
| 1. Did you have any adverse drug reactions such as allergy and nausea after taking the medicine? | | | | |
| □Not at all | □Occasionally | □About half the time | □Often | □Always |
| 1. Did you have hypoglycaemic reactions such as palpitations, dizziness and sweating? | | | | |
| □Not at all | □Occasionally | □About half the time | □Often | □Always |
| 27. How restrictive was dietary control on your lifestyle or habits? | | | | |
| □Not at all | □Somewhat restrictive | □Restrictive | □Very restrictive | □Extremely restrictive |

**Detailed Descriptions of Scales**

1. The Diabetes-specific Quality-of-Life (D-QoL)

The D-QoL was widely used to assess the QoL of T2D patients [1]. The scale has four dimensions (physiology, psychology, social, and therapy) and 27 components in the Chinese version and it has been tested for reliability and validity [1]. Scores for the responses to each item range from 1 (very satisfied) to 5 (very dissatisfied); the items are scored in reverse order. We converted the scores for each item to a positive score to facilitate the interpretation of the results; this score was obtained by adding 1 to the difference between the maximum and reverse score (Table 1).

2. The Summary of Diabetes Self-Care Activities (SDSCA)

Toobert et al. created the SDSCA tool to assess SM in T2D patients. This study used the SDSCA with 6 dimensions (11 items): food management, foot care, glucose monitoring, exercise management, and medication management [2]. The response to each item indicates the number of days the patient performed the behavior during the previous week. SDSCA is currently the most widely used and authoritative scale to measure the SMB of diabetic patients. The Chinese version of the SDSCA scale was utilized in this study. Qiao et al. localized and tested the original SDSCA scale, and the internal consistency reliability of the Chinese version of the SDSCA scale was 0.918 [3].

3. Self-Efficacy for Managing Chronic Disease 6-Item (SECD6)

The SECD6 is a widely used instrument for evaluating confidence in managing chronic disease. The reliability and validity of this scale are better after Chineseization [4]. It is made up of six items. Responses to each item range from 1 (not at all confident) to 10 (completely confident) [5]. Higher scores imply higher levels of self-efficacy.

4. The Chronic Illness Resources Survey (CIRS)

The CIRS is a well-established measure for evaluating multiple social support in individuals with chronic diseases [6]. In 2014, abbreviated version of CIRS translated and introduced in China [7]. The CIRS is rated on a Likert 5-point scale, with 1 indicating “not at all” and 5 indicating “very much;” higher scores imply more adequate social support. In this study, social support was measured using three sub-scales: physician/health care team, family/friends, and neighborhood/community.

5.The Audit of Diabetes Knowledge (AD-knowl)

The AD-knowl is a validated instrument that assesses diabetes-related knowledge. It has been localized and tested in China [8]. Each question has three response options: “correct,” “inaccurate,” and “don't know,” with a correct answer worth one point and an incorrect or “don't know” answer earning no points. This study utilized the percentage score.

6. The Problem Areas in Diabetes Scale (PAID)

The PAID is well-recognized tool for measuring diabetes-related distress [9]. The Chinese short version of the Diabetes Questionnaire has been tested for reliability and validity [10]. The tool has 20 questions ranked on a five-point Likert scale (0 for “no problem” and 4 for “serious problem”). Total scores are calculated by summing the scores, with higher scores indicating more serious issues.

The EuroQol Visual Analogue Scale (EQ-VAS)

7. The EQ-VAS is widely utilized to evaluate patients’ self-reported health. Respondents rate their health state from “0” to “100,” with “100” being the “best imaginable health status” and “0” denoting the “worst conceivable status” [11].

**Reference：**

1. Liao Z, Zhou F, Liang Y. Quality of Life Is an Important Indicator for Evaluating Diabetes Treatment. Chinese Journal of Diabetes. 2000; :49–50.

2. Toobert DJ, Hampson SE, Glasgow RE. The summary of diabetes self-care activities measure: results from 7 studies and a revised scale. Diabetes Care. 2000; 23: 943–950.

3. Qun Wang, Qiaoqin Wan, Shaomei Shang. Correlation of Self-management Behavior and Depression in Patients with Type 2 Diabetes, [J].Journal of Nursing (In Chinese),2009,24(07):14-15.

4. Yun Tong, Ze-wen Lin, Ying-wen Li, Jian-zhen Lan, Yu-mei Chen, Influence of Self-efficacy Training on Nutrition of Patients with Maintenance Hemodialysis(In Chinese). Journal of Nursing(China). 2015,22(20),47-49

5. Hu, H., Li, G., & Arao, T. (2015). Validation of A Chinese Version of the Self-efficacy for Managing Chronic Disease 6-item Scale in Patients with Hypertension in Primary Care. Isrn Public Health, 2013, 1-6.

6. Glasgow RE, Toobert DJ, Barrera M Jr, Strycker LA. The Chronic Illness Resources Survey: cross-validation and sensitivity to intervention. Health Education Research. 2005; 20: 402–409.

7. Hui-qin Zhong, Ling Fan, Ya Shao. Reliability and Validity of Chronic Illness Resources Survey (Chinese Version) in Patients with Diabetes (In Chinese). Chinese General Practice, 2014, 17(23):2779-2782.

8. Zhu W. Translation the Audit of diabetes’ knowledge and survey in 3A Hospitals in Hangzhou diabetic population (In Chinese). Master. ZhejiangUniversity;2010.

9. Eigenmann CA, Colagiuri R, Skinner TC, Trevena L. Are current psychometric tools suitable for measuring outcomes of diabetes education? Diabetic Medicine. 2009; 26: 425–436.

10. Liping Yang, Zhonghua Hu. Reliability and validity test of Short-Form Chinese version PAID scale used for elderly patients with type 2 diabetes (In Chinese). Chinese General Practice Nursing. 2015,13(20):1909-1911

11. EQ-5D User Guides – EQ-5D. https://euroqol.org/publications/user-guides/. Accessed 22 May 2023.
